# Johan Turi’s animal, mineral, vegetable cures and healing practices: an in-depth analysis of Sami (Saami) folk healing one hundred years ago

**DOI:** 10.1186/1746-4269-9-57

**Published:** 2013-08-13

**Authors:** Thomas A DuBois, Jonathan F Lang

**Affiliations:** 1 University of Wisconsin-Madison, Madison, USA

## Abstract

**Background:**

The healing knowledge of a Sami (Saami) hunter and reindeer herder was surveyed as a window into the concepts of health, healing, and disease in early twentieth-century Sapmi (Northern Sweden). The two books of Johan Turi (1854–1936)—*An Account of the Sami* (1910) and *Lappish Texts* (1918–19) were examined to determine the varieties of recorded zootherapeutic, mineral, chemical, and ethnobotanical lore, as well as the therapeutic acts, identified conditions, and veterinary knowledge included.

**Methods:**

Tabulation of the materials and species mentioned in Turi’s descriptions (n = 137) permitted analysis of the relative frequency of differing types of healing in Turi’s overall therapeutic repertoire, his relative attention to chronic vs. acute ailments, and the frequency of magic as a component of healing. A qualitative appraisal was made of the degree to which outside influences affected Sami healing of the period. A further assessment of the possible clinical efficacy of the recorded remedies was undertaken.

**Results:**

Turi’s remedies consist most often of zootherapeutics (31%), followed by physical acts such as massage, moxibustion, or manipulation (22%). Ethnobotanical cures make up a significantly smaller portion of his repertoire (17%), followed by mineral and chemical cures (12%). Magic rituals (including incantations and ritual acts) make up a significant portion of Turi’s repertoire, and could be used alone (17%) or in conjunction with other types of healing (38%). Turi’s healing aimed primarily at acute ailments (65%), with chronic conditions addressed less often (35%). A literature review revealed that Turi’s remedies held a marked frequency of likely efficacy, at least in cases in which it was possible to ascertain the precise species, conditions, or substances described. Although it is possible at times to recognize foreign sources in Turi’s repertoire, it is clear that Turi understood all his healing methods as distinctively Sami.

**Conclusion:**

The research illustrates the variety and depth of a single informant’s healing knowledge, and demonstrates the value of both historical sources and in-depth data collection with single experts as useful means of assessing and characterizing an indigenous population’s healing traditions.

## Background

As the indigenous population of Fennoscandia, Sami people (also called Saami, formerly called Lapp) developed a wide range of traditional medical knowledge and practices related to successful living in Europe’s far north. Negotiating the region’s harsh weather and daylight cycles and comparatively limited food resources placed a considerable burden on Sami people in terms of health. By the early twentieth century, most Sami lived off a combination of hunting, gathering, reindeer husbandry, and occasional small-scale farming. Millennia of close contact with neighboring Nordic and Slavic peoples had led to the incorporation of medical lore from elsewhere, and sometimes, as medical regimes changed over time, practices once common throughout the region were retained in northern peripheral communities but replaced elsewhere. The Sami hunter, trapper, and sometime reindeer herder Johan Turi (1854—1936) provided a detailed snapshot of Sami medical knowledge during this period through the text he wrote, *Muitalus Samiid birra*[1] (Turi 1910; translated as *An Account of the Sami,* 2011)—the first secular book ever written in Sami language. Turi wrote this work in collaboration with a Danish ethnographer and artist, Emilie Demant Hatt (1873—1958), who produced the first translation of the work into Danish and edited it for initial publication. He also produced further materials regarding healing that he refused to allow Demant Hatt to publish in his 1910 work, although these eventually became available to readers through a second volume, *Lappish Texts*[2] (Turi 1918–19). Examining these materials a century later can provide valuable indications of cultural continuities and changes within Sami healing traditions.

The authors of the present study set out to examine Turi’s medical knowledge in order to survey the main categories of Sami healing lore at the opening of the twentieth century and to assess the possible clinical efficacy of Sami healing traditions. Turi’s compendium of traditional knowledge and narratives provides a glimpse of the rich healing knowledge that a single, competent healer in Sami society commanded in the early twentieth century, and also furnishes important historical data for possible future comparisons with Sami healers of today. Although Turi’s work is comparatively well known within the study of Sami culture, it has only recently been accurately translated into English [1] and, apart from some general studies of Sami folk medicine, has never been systematically examined as a holistic source of Sami medical knowledge.

Johan Turi was born in 1854 in Guovdageaidnu (Kautokeino), Norway, into a prominent and comparatively wealthy family of reindeer herders. Like many Sami of the era, however, Turi’s family eventually lost access to key grazing lands that made it possible for the family to maintain a sizeable herd of reindeer which they used for meat, milk, fur, and antler. In order to maintain their threatened livelihood, the family relocated to Gárasavvon (Karesuando), Sweden, in 1857, and again to Čohkkeras (Jukkasjärvi), Sweden, some two decades later. Turi participated in the family’s migratory herding life, but eventually gave up reindeer husbandry for a life of hunting and trapping. Because he was often on his own in this latter livelihood, Turi became proficient in Sami healing practices, and he seems to have developed a local reputation as a skilled healer among his community. Turi also attributed much of his knowledge to his father and grandfather, who had also been known as skillful healers in their day.

Although Turi’s decision to create a book seems to have been his own, his intention was made a reality largely through the assistance and friendship of Emilie Demant Hatt. Their collaboration signaled a new approach to ethnography, in which native informants began to be accorded respect and status as authors or co-authors of the scientific studies that drew on their knowledge (see for example, the collaboration of Kristoffer Sjulsson and O. P. Petterson [3], Anta Pirak and Harald Grundström [4], and Iam Saem Majnep and Ralph Bulmer [5]). Given their close working relations, it is natural that Demant Hatt’s interests played a significant role in shaping the content and details of Turi’s text [6], particularly in the area of healing. Turi supplied her with more information than he was at first willing to see in print, including detailed instructions about the use of magic formulas and “case histories” of healing events that he had witnessed in his life. Much of Turi’s more secret knowledge came to print only in his later volume, but all of it was recorded in part because of Demant Hatt’s strong interest in the topic. The current study examines both of Turi’s published volumes [1,2] on this topic in terms of the healing knowledge they contain.

Turi wrote his text at a crucial time of transition for Sami people. At least a century of large scale in-migration of other peoples—Finns, Swedes, Norwegians—had introduced new healing traditions and methods into local use. In addition, a state-supported medical doctor had become established within easy traveling distance of Jukkasjärvi, making then-current Western medicine available to Sami for the first time [7]. These factors make Turi’s compendium particularly interesting for the cross-cultural analysis of indigenous healing traditions within situations of massive cultural change.

## Methods

In order to assess Johan Turi’s medical knowledge within its cultural and historical contexts, the researchers tabulated all healing procedures and substances mentioned in either of the above-mentioned texts. As such, the study represents an extended literature review and analysis and entailed no human experimentation or first-hand human subjects research of any kind.

The organization of Turi’s works can appear haphazard to the present-day reader, and in any case, Turi did not set out to present his knowledge in anything like the manner of a medical manual of today. Healing instructions are sometimes grouped in particular sections having to do with affected body parts or the animal species from which zootherapeutic remedies are derived, but they may also occur in passing, when Turi is describing larger tasks of Sami life, such as reindeer herd migration, trapping, and food preparation. Turi’s organization of his material represents a rich source of cultural information in itself, as we discuss below, but it can render comparative research with other healing traditions difficult. Once this tabulation of data was complete, the researchers were able to analyze various overarching factors reflected in Turi’s material:

1) the relative frequency of differing categories of healing within Turi’s overall body of medicinal knowledge;

2) the relative frequency of treatments for acute and chronic conditions;

3) the potential clinical efficacy of Turi’s remedies;

4) the degree to which magic is used in Turi’s remedies;

5) the degree to which Turi’s material reflects uniquely Sami knowledge or shows the influences of neighboring cultures and medical traditions at the outset of the twentieth century.

## Results

The references cited within the tables presented here direct the reader to either, A, mentions of similar remedies used in other cultures; B, historical commentaries on a particular remedy or technique; or C, research that evaluates the possible efficacy of compounds or techniques described in the remedy. Where appropriate, the researchers have also examined plant remedies in relation to other plant species that were important to the Sami diet during the early twentieth century.

For clarity and simplicity, remedies from the source *Muitalus Samiid birra* are designated “T1,“ followed by the corresponding page number. Remedies from *Lappish texts* are designated “T2” followed by the corresponding roman numeral for that particular section of the published text.

I. Zootherapeutics (Table 1)

**Table 1:** Zootherapeutic remedies

**Species**	**Source**	**Use(s)**	**Method**	**References**
*Canis lupus (*wolf)	T1 p. 107	gout, body aches, blisters, wounds	rub fat directly on affected area	B: [8,9]
				C: [10]
	T1 p. 107	wounds, wolf bite	pour gall on wound, wrap with adipose tissue	A: [11,12]
				C: [11]
*Canis lupus familiaris* (dog)	T1 p. 127	dog bite	rub blood of dog on wound*	
	T2 XXXVII	pregnancy cravings	pass partially chewed food to a female dog*	A: [12,13]
				B: [14]
				C: [15,16]
	T2 II	recovering from various diseases	the presence of a dog will draw away illness*	B: [14]
*Delichon urbicum* (house martin)	T1 p. 125	hemorrhage during childbrith	drink house martin nest litter boiled in milk**	A: [12,17,18]
				C: [19-23]
*Dytiscus sp.* (diving beetle)	T1 p. 123	causes fatal illness if swallowed	use a straw made from reindeer antler or bird bone to drink water from streams, if swallowed induce vomiting with rotten fish entrails, or a reindeer tendon	
*Homo sapiens* (human)	T1 p. 121	sore throat	drink a spoonful of urine and stretch and rub neck in every direction	A: [12,13]
				C: [24]
	T1 p. 128	difficult labor	have the mother drink the urine of the father and say his name**	A: [12,13]
				C: [24]
	T2 XVI	unreciprocated love	have the person consume some of your sweat*	C: [25-28]
	T2 XVI	unreciprocated love	have the person consume a couple drops of your blood*	
	T2 XVI	unreciprocated love	have the person consume a few scales from your foot*	
*Ovis aries* (sheep)	T1 p. 121	sore throat	rub turpentine on neck and then wrap in a woolen kerchief overnight	A: [12,29]
				B: [30,31]
				C: [32,33]
	T1 p. 125	strained tendon	wrap affected limb in unwashed woolen yarn**	A: [12,13,29]
	T1 p. 129	inability to pass afterbirth	place a hot compress of sand and ash wrapped in woolen fabric just below mother’s chest	A: [12,13,29]
				C: [20]
*Pediculus humanus* (lice)	T1 p. 126	jaundice	secretly feed the affected person nine lice in buttered frybread or gruel*	A: [12,13,34,35]
*Rana temporaria* (frog)	T1 p. 122	sore throat	find a frog with white markings, dry in a saltbin, cut into pieces, cook in milk, and then drink	A: [12,36]
				C: [37-45]
	T1 p. 122	skin eruptions	rub a frog with white markings directly on the affected area**	"
	T1 p. 122	healing hand	catch a frog and have it urinate on your hand, the hand can be used to relieve pain*	"
	T1 p. 123	thrush	press a frog with white markings on the tongue	"
	T1 p. 123	stomach ailments	cook a frog with white markings in milk and drink	"
	T1 p. 123	eggs cause fatal illness if swallowed	if eggs are swallowed induce vomiting with rotten fish entrails, or a reindeer tendon	
*Rangifer tarandus* (reindeer)	T1 p. 22	healthy drink	add reindeer milk and sorrel (*Rumex acetosa*) to hot water	A: [12,46]
	T1 p. 24	nutrition for baby when no milk is available	give baby reindeer fat to suck on	
	T1 p. 55	stomach ailments	boil reindeer brains with pine bark and fat and then ingest	A: [12]
				C: [47]
	T1 p. 123	induces vomiting	force a tendon from a reindeer’s leg down the throat	
	T1 p. 124	swelling	rub reindeer fat (sometimes mixed with flecks of copper) onto swelling	A: [12]
				B: [8,48]
				C: [49,50]
	T1 p. 125	chills	drink reindeer blood	
	T1 p. 126	sore tooth or gland	press a heated reindeer jawbone on the affected area*	B: [14]
	T1 p. 127	wounds	rub reindeer cheese on the affected area	A: [12]
	T1 p. 128	inability to pass afterbirth	give woman reindeer butter to eat	
	T1 p. 129	inability to pass afterbirth	grind up downy birch buds (*Betulina pubescens*), mix with hot reindeer milk, sorrel, and water, and give to mother to drink	A: [12]
				C: [20,51]
	T1 p. 169	burns	apply reindeer bone marrow to affected area	A: [12]
				C: [52]
	T2 XXXVI	sprained tendon	wrap a reindeer tendon around the affected limb	A: [12]
*Ursus arctos* (brown bear)	T1 p. 98	heart problems and internal ailments	drink bear gall	A: [11,12]
				C: [11,53-55]
	T1 p. 98	wounds	pour bear gall on affected area	"
	T1 p. 98	throat rash	pour milk through a bear trachea three times and then drink the milk*	A: [12]
				B: [14]
	T1 p. 98	gout, body aches, blisters, wounds	cover affected area in bear fat	A: [12]
				B: [9,11]
				C: [10]
	T1 p. 126	sore tooth or gland	press a bear tooth on the affected area	A: [12,13,56]
				B: [14]
*Miscellaneous*				
Rotten fish entrails	T1 p. 123	swallowed frog eggs or diving beetle	give rotten fish entrails to a person for ingestion, which will induce vomiting	
Animals and birds for divination	T1,T2 p. 111–112, XI	prediction of weath, luck, impending death	examining patterns in bird and animal behavior*	A: [13,29,56]
Snakestone	T1 p. 115	maintaining good luck in legal matters	steal a snakestone*	A: [12,29,56]

*magical treatment.

**accompanying incantation.

A: mentions of similar remedies used in other cultures.

B: historical commentaries on a particular remedy or technique.

C: research that evaluates the possible efficacy of compounds or techniques described in the remedy.

II. Ethnobotanical lore (Table 2)

**Table 2:** Botanical remedies

**Species**	**Source**	**Use(s)**	**Method**	**References**
*Angelica archangelica* (wild celery)	T1 p. 54	milk stabilizer and supplement	add young plants (Sami: fadno) to milk	A: [12,13]
				B: [57-59]
	T1 p. 54	food source	flavor old plants (Sami: *boska*) with salt and eat	A: [12,13]
				B: [57-59]
*Betula pubescens* (downy birch)	T1 p. 125	itchy skin	rub affected area with ashes	A: [12]
				B: [58]
				C: [51]
	T1 p. 129	inability to pass afterbirth	grind up downy birch buds (*Betulina pubescens*), mix with hot reindeer milk, sorrel, and water, and give to mother to drink	A: [12]
				B: [58,59]
				C: [20,51]
*Betula* sp.(birch)	T1 p. 54	coffee preparation (a drink)	use a bracket fungus, grain, and birch sap (*Betula* sp*.*)	B: [58-60]
	T1 p. 124	swelling and drawing out pus from a boil	apply a thin layer of birch bark (*Betula* sp.) to the affected area	A: [12]
				B: [58]
*Coffea arabica* (coffee)	T1 p. 121	headaches	massage head and neck, pull hair at the apex of the head, wash the head in hot coffee	A: [12]
				C: [61]
*Ferula assafoetida* (stinking assa)	T2 XXXIX	parasites in dogs	give the dog stinking assa (*Ferula assafoetida*) and sulfur to eat	A: [12,62,63]
				B: [62]
				C: [62,64-66]
Lichen (sod)	T2 XVIII	earth *bostta*	rub a piece of sod (possibly including lichens) on affected area	B: [58]
				C: [67,68]
(*Usnea* sp.)	T1 p. 54	bread preparation	use beard lichen (*Usnea sp.*), other lichens, inner bark of a pine tree (*Pinus sylvestris*), with a little flour added	B: [58-60]
				C: [47]
*Pinus sylvestris* (Scots pine)	T1 p. 54	bread preparation	use beard moss (*Usnea* sp.), lichens, inner bark of a pine tree (*Pinus sylvestris*), with a little flour added	A: [12]
				B: [58-60]
				C: [47]
	T1 p. 55	stomach ailments	boil reindeer brains with pine bark (*Pinus sylvestris*) and fat and then ingest	A: [12,69]
				B: [58-60,70]
				C: [47]
*Piptoporus betulinus* (birch polypore)	T2 XXXV	toothache, fractures, rheumatism, headache, pneumonia	burn a small amount of fungus (*Piptoporus betulinus*) directly on the affected area	A: [12,71]
				B: [58]
				C: [72]
*Rumex acetosa* (sorrel)	T1 p. 22	healthy drink	add reindeer milk and sorrel (*Rumex acetosa*) to hot water	A: [12,69]
*Taphrina betulina* (witch’s broom)	T1 p. 125	itchy skin	boil witch’s broom (*Taphrina betulina*) in water and then rub the preparation on the affected area	A: [12]
				C: [51,73]
*Miscellaneous*				
leaves from nine different kinds of trees	T1 p. 125	itchy skin and many other ailments	boil all leaves together and apply the mixture to the affected area	B: [58]

*magical treatment.

**accompanying incantation.

A: mentions of similar remedies used in other cultures.

B: historical commentaries on a particular remedy or technique.

C: research that evaluates the possible efficacy of compounds or techniques described in the remedy.

III. Minerals and chemicals (Table 3)

**Table 3:** Mineral and chemical remedies

**Compound**	**Source**	**Use(s)**	**Method**	**References**
*alcohol*	T1 p. 58	Turi says it is a substance which causes great harm	ingestion	
*ash/sand*	T1 p. 129	inability to pass afterbirth	place a hot compress of sand and ash wrapped in woolen fabric just below mother’s chest	A: [12,13,29]
				C: [20,33]
*copper*	T1 p. 124	swelling	mix flecks of copper into reindeer fat and apply to swelling	A: [12,13]
				B: [8,48]
				C: [49,50]
*mercury* (quicksilver)	T1 p. 127	broken bones, contusions, shooting pains, severe diarrhea	swallow a spoonful of quicksilver**	A: [12,13,56]
				B: [74]
				C: [74,75]
	T1 p. 127	sties and other eye ailments	apply quicksilver to affected area	"
	T1 p. 127	protects against ghosts	carrying quicksilver*	A: [12,56]
*Muscovite* (fox gold, yellow mica)	T1 p. 128	joint problems	grind the muscovite as fine as flour, mix in water, and then drink*	B: [76]
				C: [77]
*silver*	T1 p. 120	excessive bleeding	press the bleeding vessel with a silver coin*	A: [12,13]
	T1 p. 170	pain relief	press silver to affected area*	A: [12,13]
				B: [14]
*snakestone*	T1 p. 115	skill at law	steal a snakestone from where snakes breed*	A: [12,29,56]
*soot* (carbon or copper oxides)	T1 p. 124	male urinary blockage	rub soot from the bottom of a copper kettle on the outside of the penis	A: [12,29]
*strychnine*	T1 p. 94	wolf poison	put strychnine mixed with lead shavings or inside a tallow plug in chunks of reindeer meat	
*sulfur*	T2 XXXIX	parasites in dogs	give the dog stinking assa (*Ferula asafetida*) and sulfur to eat	A: [13]
				C: [78]

*magical treatment.

**accompanying incantation.

A: mentions of similar remedies used in other cultures.

B: historical commentaries on a particular remedy or technique.

C: research that evaluates the possible efficacy of compounds or techniques described in the remedy.

IV. Conditions (Table 4)

**Table 4:** Conditions

**Ailment**	** Source**	**Method**	**References**
appetite loss	T1 p. 119	let blood from above the foot*	A: [12,13]
			B: [79]
backaches	T1 p. 126	massage the area, pull and stretch the skin	A: [12,13,29]
			C: [80,81]
bleeding, hemorrhage	T1, T2 p. 129, XXV, XXXI	recite an incantation**	A: [29]
body aches (hip, back, chest, shoulder, shooting pains)	T1, T2 p. 119, XXVIII	let blood from specific areas of the body*	A: [12,13]
			B: [79]
boils	T2 XXIX	recite an incantation**	A: [13,29]
*bostta*	T1, T2 p. 122, 128, XVIII, XIX	(see below)	A: [12]
dead body *bostta* (from touching a dead person’s clothing or smelling their corpse)	T2 XVIII	recite an incantation**	A: [13,29]
dead body *bostta* (from ingestion of corpse fluid)	T2 XVIII	none provided	
dry-earth *bostta* (psoriasis?)	T2 XVIII	rub a piece of sod (possibly a lichen) on affected area**	C: [67,68,82]
earth *bostta* (infectious disease?)	T2 XVIII	rub a piece of sod (possibly a lichen) on affected area**	C: [67,68,82]
old-maid *bostta* (unknown)	T2 XVIII	press the sick spot with the old-maid’s clothes and recite an incantation**	B: [14]
wet-earth *bostta* (small pox, chicken pox, eczema?)	T2 XVIII	rub a piece of sod (possibly a lichen) on affected area**	C: [67,68,82]
wind *bostta* (genetic condition, maybe eczema?)	T2 XVIII	recite an incantation**	
chest pain	T1 p. 119	let blood from above the foot*	A: [12,13]
			B: [79]
childbirth	T1, T2 p. 24, 128–129 XXXVIII	advice and various techniques provided (see other tables)**	A: [12,13]
constipation	T1 p. 124	administer an edema of oatmeal and warm water, or reindeer bone fat and warm water	A: [12,13]
coughing	T1 p. 126	cool soles of patient’s feet with ice and then heat them up as hot as the patient can stand	A: [12,13,63]
dislocated joints and fractures	T1 p. 167	cool the affected bones in a stream until numb then set the bones to the proper positions, secure with a splint	A: [13]
drowning	T1 p. 127	lay victim so water can drain out of the lungs, be quiet so as to not frighten the life spirit, massage the victim	A: [83]
			C: [84]
fainting	T1 p. 120	burn undergarments, let blood from nine different locations*	
	T1 p. 127	flap victim’s arms up and down	A: [83]
			C: [85]
fractures	T2 XXXV	burn a small amount of fungus (*Piptoporus betulinus*) directly on the affected area	A: [12]
frostbite	T1 p. 124	massage affected area with subsurface snow until it becomes red again	A: [12]
gout	T1 p. 120	let blood from the outer side of the leg, arm, and ankle	A: [12,13]
			B: [79]
headache	T1 p. 121	massage head and neck, pull hair at the apex of the head, wash the head in hot coffee	A: [12,13,29]
			C: [61,81]
	T2 XXXV	burn a small amount of fungus (*Piptoporus betulinus*) directly on the affected area	A: [12]
nausea	T1 p. 127	measure the belt of the affected person (to induce vomiting)	A: [13]
pneumonia	T2 XXXV	burn a small amount of fungus (*Piptoporus betulinus*) on body	A: [12]
pregnancy cravings	T2 XXXVII	pass partially chewed food to a female dog*	A: [12]
			B: [14]
			C: [16]
rheumatism	T2 XXXV	burn a small amount of fungus (*Piptoporus betulinus*) directly on the affected area	A: [12]
startling	T1 p. 120	bleed three small amounts of blood from the “heart artery,” if bleeding persists, press with silver coin*	A: [12,13,29]
			B: [79]
strained spermatic cords	T1 p. 126	rub gently in an upward direction	A; [12]
			C: [80,81]
strained tendons	T2 XXVI	wrap with wool and recite an incantation**	A: [12]
			C: [33]
swelling	T1 p. 124	mix flecks of copper into reindeer fat and apply to swelling	B: [8,48]
			C: [49,50]
	T1 p. 124	apply a thin layer of birch bark (*Betula* sp.) to the affected area	A: [12]
	T2 XXXIV	burn a small piece of sailcloth on affected area	A: [12]
swelling explanation	T2 XXI, XXVII	an invasion of cold, heat, water, or perspiration into a wound, can use magic to prevent*	B: [7]
toothache	T1 p. 121	stab the sore gland near tooth with an awl, release at least three drops of blood	A: [12]
			C: [86]
toothache	T1 p. 126	rub both sides of throat, all around the mouth, neck tendons, and back tendons	C: [61,86]
toothache	T2 XXXV	burn a small amount of fungus (*Piptoporus betulinus*) directly on the affected area	
unconscious infant (strangled by umbilical cord)	T1 p. 24	suck on mouth and nose, leave cord intact	
urinary stoppage (female)	T1 p. 124	administer an edema of oatmeal and warm water, or reindeer bone fat and warm water	A: [12]
urinary stoppage (male)	T1 p. 124	rub soot from the bottom of a copper kettle on the outside of the penis	A: [12]
wound caused by iron	T2 XXXI	recite an incantation**	A: [29]

*magical treatment.

**accompanying incantation.

A: mentions of similar remedies used in other cultures.

B: historical commentaries on a particular remedy or technique.

C: research that evaluates the possible efficacy of compounds or techniques described in the remedy.

V. Treatment regimens (Table 5)

**Table 5:** Treatment regimens

**Method**	** Source**	**Use(s)**	**Method summary**	**References**
bloodletting	T1 p. 119	aches and pains of hip, back, chest, headache, shoulder, and acute shooting pains in other parts of the body	bloodletting at specific locations*	A: [12,13]
				B: [79]
cupping	T1 p. 120	for various sorts of headache, back and chest pain, toothaches, and sore legs	cupping at specific locations using an animal horn*	A: [12,13,29,56]
divination	T1, T2 p. 111–112, XI	prediction of weath, luck, impending death	divination by examining bird and animal behavior*	A: [13,29,56]
	T1 p. 135	to see what is happening at different places	divination by looking into alcoholic beverages*	A: [13,29,56]
edema	T1 p. 124	constipation, urinary stoppage	application of an edema made from either oatmeal and warm water or reindeer bone fat and warm water	A: [12,13]
lancing	T1 p. 121	toothache	stab the sore gland near tooth with an awl, release at least three drops of blood	A: [12]
magic	T2 L	preventing misfortune	recite an incantation**	A: [13,29,56]
magic (harm)	T1, T2 p. 133–137,III, V-XIV, XLXLVII, LIV, LV	invoking supernatural aggression	recruit the help of a *noaidi* spirit worker*	
magic (ingestion of human subtances)	T2 XIII, XVI, XLVIII	unreciprocated love	secretly give a small amount of blood, skin, or sweat to a person to eat to make them fall in love*	A: [29]
				C: [25-28]
magic (object)	T1 p. 115	maintaining good luck in legal matters	steal a snakestone*	A: [12,29,56]
magic (offerings)	T1 p. 13, 111, 134	maintaining good luck	leave offerings of reindeer carcasses, fish fat, and other precious objects at sacred sites*	A: [13,29,56]
magic (offerings)	T1, T2 p. 85, 156, 159,167, I-III	maintaining good luck	leave offerings of brass, gold, or silver coins at sacred sites, or pour portions of coffee or liquor into the ground*	A: [13,29,56]
magic (words and transference)	T2 XXIX	boils	recite an incantation and press a key on top of the boil**	A: [13,29,56]
				B: [14]
magic (words)	T2 XXXI	wounds caused by an iron weapon	recite an incantation to accelerate healing**	A: [13,29,56]
	T1, T2 p. 125, XXII	many ailments	recite an incantation, however, not many people are skilled in this area**	A: [13,29,56]
	T1 p. 125	hemorrhage in chilbirth	recite an incantation along with the administration of bird nest litter boiled in milk**	A: [17,18]
				C: [19-23]
	T1 p. 128	abscesses and tumors	recite an incantation**	A: [13,29,56]
	T1 p. 128	difficult labor during childbirth	have the mother say the father’s name and drink some of his urine**	C: [24]
	T1 p. 44	prevention of bewitchment on Christmas Eve	recite sections of the Bible by heart**	A: [13,29,56]
	T1 p. 104	driving wolves away	cut a square out of a fresh wolf snow print and recite an incantation**	
	T1 p. 122	skin eruptions	recite an incantation while rubbing a frog on the affected area**	A: [36]
				B: [41]
				C: [36-40,42-45]
	T2 XV	exorcising ghosts	recite an incantation**	A: [13,29,56]
	T2 XVIII, XIX, XXIII	treating bostta	recite an incantation**	"
	T2 XXI	swelling	recite an incantation**	"
	T2 XXX	contusions	recite an incantation**	"
	T2 XXV	staunching bleeding	recite an incantation**	"
	T2 XXVI	strained tendons	recite an incantation**	"
	T2 XXVIII	body aches	recite an incantation**	"
massage	T1 p. 124	frostbite	rub the affected area with subsurface snow (“corn snow”)	A: [12,13,29]
	T1 p. 126	strained spermatic cords	rub gently in an upward direction	A: [12]
				C: [80,81]
	T1 p. 126	backache	massage the area, pull and stretch the skin	A: [12,13,29]
				C: [80,81]
	T1 p. 126	toothache	rub both sides of throat, all around the mouth, neck tendons, and back tendons	A: [12,29]
				C: [61,86]
moxibustion	T2 XXXIV	swelling	burn a small piece of sailcloth on the affected area	A: [12]
	T2 XXXV	toothache, rheumatism, fracture,headache, pneumonia	burn a small amount of fungus (*Piptoporus betulinus*) directly on the affected area	A: [12,71,72]
physical manipulation	T1 p. 167	dislocated joints and fractures	cool the affected bones in a stream until numb then set the bones to the proper positions, secure with a splint	
poisoning	T2 XXXIII, XLVI	source of poison	collect fluids from a dead body and use as an oral poison	
resuscitation	T1 p. 24	unconscious infant (strangled by umbilical cord)	suck on mouth and nose, leave cord intact	
	T1 p. 127	fainting	flap victim’s arms up and down	A: [83]
				C: [85]
transference	T2 XXXV	toothache	press a bear’s tooth or reindeer jawbone on the affected area*	A: [12,13,29,56]
				B: [14]
	T2 XXXVII	pregnancy cravings	pass partially chewed food to female dog*	A: [12,13,29]
				B: [14]
				C: [16]
vomition	T1 p. 123	after having swallowed frog eggs or a diving beetle	use rotten fish entrails or a reindeer tendon to induce vomiting	

*magical treatment.

**accompanying incantation.

A: mentions of similar remedies used in other cultures.

B: historical commentaries on a particular remedy or technique.

C: research that evaluates the possible efficacy of compounds or techniques described in the remedy.

As indicated in Table 5, another way to look at Turi’s healing knowledge is through reference to particular regimens of treatment that he describes as particularly effective in healing a variety of ailments. Many of these derive ultimately from foreign sources, but are viewed by Turi as specifically Sami. Given that they had been largely abandoned in the official medicine now offered through the medical office at Vittangi [87] it is understandable that Turi saw these regimens as notably Sami.

VI. Veterinary medicine (Table 6)

**Table 6:** Veterinary remedies

**Species**	**Source**	**Disease**	**Method**	**References**
*Rangifer tarandus* (reindeer)	T1 p. 31	dieigečalbmi: eyes turn white, can cause blindness, exacerbated by insects (keratitis?)	place a louse and sometimes sulfur in the affected eye	A: [13]
				C: [88-90]
	T1 p. 31	no Sami name given: circling disease (listeriosis?)	boil bark in water until it is as thick as tar, then smear on the affected area	C: [47,88-90]
	T1 p. 31- 32	ruodnu: reindeer walks around like it is about to urinate but nothing comes out, pus in urethra, bladder, and intestines (cystitis or pyelonephritis?)	boil fish oil, butter, tar, and gunpowder in water and then pour the mixture down the animal’s throat	C: [88-90]
	T1 p. 31- 32	livzzavihki: emaciated, rear end sags (parasitic infection by Elaphostrongylus rangiferi)	boil bark in water until it is as thick as tar, then smear on the affected area	C: [47,88-90]
	T1 p. 31-32	čagarvihki: swelling of reindeer penis (urolithiasis?)	boil bark in water until it is as thick as tar, then smear on the affected area	C: [47,88-90]
	T1 p. 31-32	njunnevihki: muzzle develops scabs, spreads to tongue and mouth, and then to the throat and lungs, fatal (Aphtae epizooticae “foot-and-mouth disease”?)	boil bark in water until it is as thick as tar, then smear on the affected area	C: [47,88-91]
	T1 p. 31-32	geardni: udder develops scabs and swells, eventually falling off. Scabs spread to mouth and lungs and kills the animal (Aphtae epizooticae “footand-mouth disease”?)	take hoof fat and boil it with pine or fir resin, then rub the affected areas with the mixture	C: [88-90]
	T1 p. 31-32	šlubbu: swelling and pus in hoof (infectious pododermatitis?)	boil bark in water until it is as thick as tar, then smear on the affected area	C: [47,88-90]
*Canis lupus familiaris* (dog)	T2 XXXIX	dog madness (rabies?)	remove a “worm” from under the dog’s tongue*	B: [92,93]
	T2 XXXIX	parasitic infection	give the dog stinking assa (*Ferula assafoetida*) and sulfur to eat	A: [12,62,63]
				B: [62]
				C: [62,64-66,78]

*magical treatment.

**accompanying incantaion.

A: mentions of similar remedies used in other cultures.

B: historical commentaries on a particular remedy or technique.

C: research that evaluates the possible efficacy of compounds or techniques described in the remedy.

## Discussion

Tabulation and analysis of Turi’s healing knowledge allowed the researchers to address five key analytical questions:

1) the relative frequency of differing categories of healing within Turi’s overall body of medicinal knowledge;

2) the relative frequency of treatments for acute and chronic conditions;

3) the potential clinical efficacy of Turi’s remedies;

4) the degree to which magic is used in Turi’s remedies;

5) the degree to which Turi’s material reflects uniquely Sami knowledge or shows the influences of neighboring cultures and medical traditions at the outset of the twentieth century.

### The relative frequency of differing categories of healing within Turi’s overall body of medicinal knowledge

**Figure 1:**
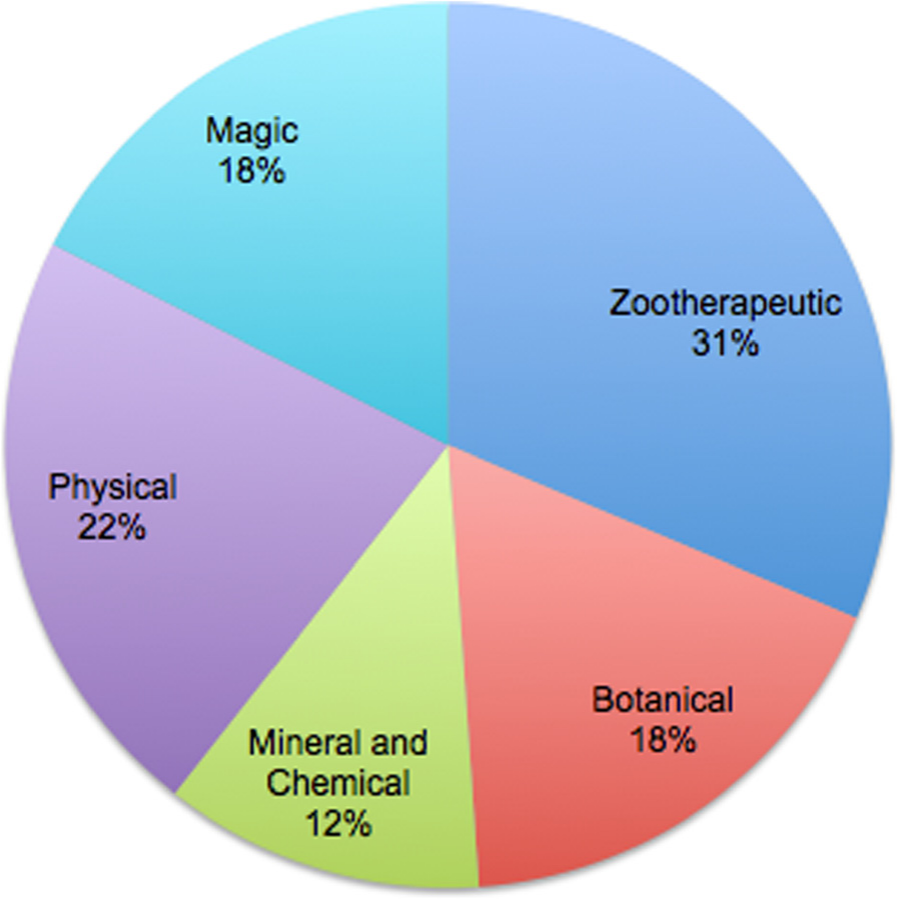
Turi’s remedies by category.

As Figure 1 shows, Turi’s healing arsenal shows a fairly even reliance on all healing categories, but a slightly higher portion of his remedies relying on zootherapeutic sources. One typically expects folk healers to rely most heavily on botanical remedies, but as demonstrated here these only comprise a small percentage of the whole of Turi’s remedies. This may reflect the reality of life at higher latitudes with its long winters, short growing seasons, and thus reduced overall plant diversity from which to discover pharmaceutical uses.

### The relative frequency of treatments for acute and chronic conditions

**Figure 2:**
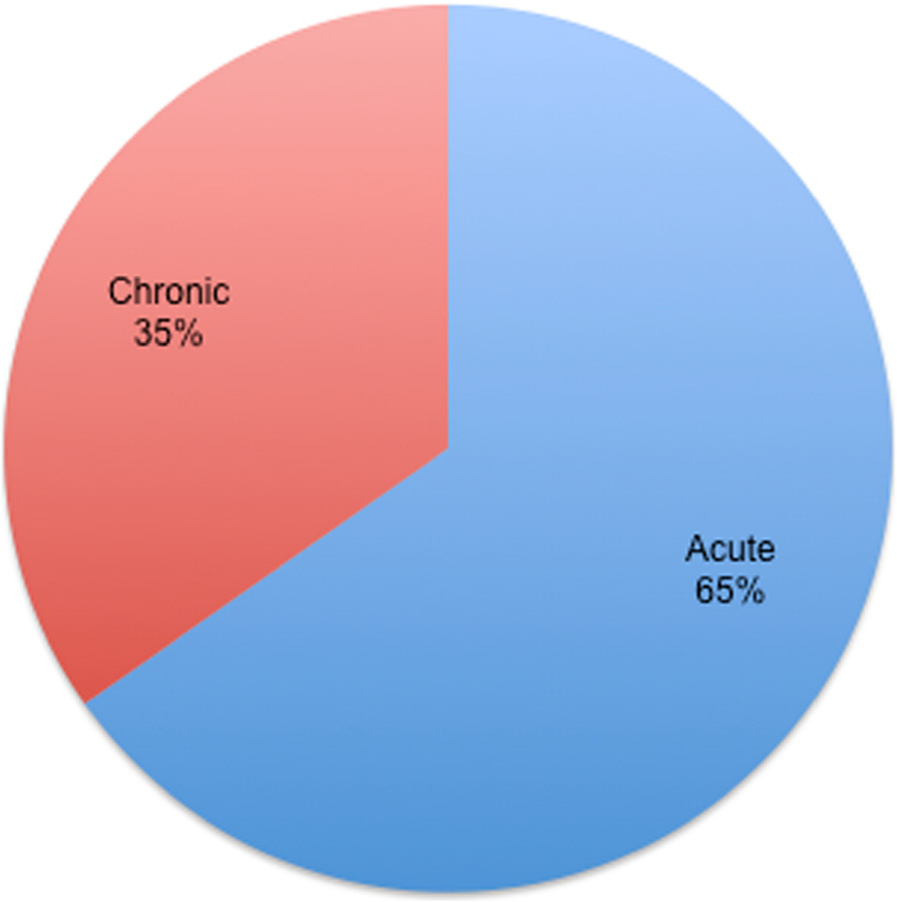
Remedies for acute and chronic conditions.

As Figure 2 shows, Turi’s compendium pays far more attention to acute ailments than to chronic conditions. This finding disproves general assumptions regarding the practice of folk healing in the Nordic region [29], in which chronic ailments have generally been identified as the more typical objects of folk healing activities. Turi’s practices may reflect the fact that recourse to “official” medicine was relatively recent in his area of northern Sweden, consisting only of a single district medical office in Vittangi [87]. Prior to the establishment of this office, Sami of Turi’s generation or earlier were obliged to heal themselves, regardless of whether the complaint was an acute ailment (for which later generations of Sami would regularly consult an official medical doctor) or chronic ailments (which remain relatively less liable to trigger a medical consultation).

### Clinical efficacy of Turi’s healing

As indicated in the overall tabulations presented below, a number of Turi’s healing methods appear confirmed by later medical and pharmaceutical research. A sample of the studies the researchers uncovered illustrate the range and nature of the kinds of cures which Turi describes.

#### Zootherapeutics

As summarized in Table 1, Turi recommends various substances derived from animals as healing agents. **Bear gall**, a substance which Turi recommends for the treatment of heart problems, wounds, and other internal ailments, is composed primarily of bile salts with a high percentage being ursodeoxycholic acid [53]. Bear bile has been used for detoxification, fever reduction, inflammation, pain relief, and swelling in traditional Chinese medicine [11]. In western official medicine, ursodeoxycholic acid (UDCA) is currently used to treat primary biliary cirrhosis, an autoimmune disease caused by inflammation and degradation of the bile ducts [54]. It should be noted, however, that treatment with UDCA can only alleviate some of the symptoms in the early stages of the disease, not cure it. Interestingly, current studies are under way to test the effects of UDCA in cardiovascular disease [55], which lends further credibility to Turi’s suggested use of the compound.

Using UDCA to treat wounds does not appear directly in current scientific literature. It is noteworthy, however, that in the instances in which Turi describes the administration of gall for wounds, he also recommends the application of a layer of fat to the surface of the wound as well. The use of animal fat to dress wounds has a clear historical precedent [8]. It is not difficult to imagine the advantages of applying fats to wounds as a means of creating a physical barrier against infection or contamination. Additionally, the fat can be used as a medium for the sustained application of other chemicals such as copper, as Turi details. The formula for a patented rash ointment suggests that the presence of animal fats with lipid compositions similar to those of human cells may accelerate cell proliferation in damaged skin [10].

The illnesses for which Turi recommends the use of **frogs***(Rana temporaria)* appear to be mostly infectious. These include thrush (a fungal infection), sore throats (possibly bacterial in origin), and stomach ailments (also possibly bacterial in origin). A study conducted on *Rana temporaria* in Russia was in part motivated by the report that native populations in northern Russia and Finland put frogs in milk vessels to delay milk souring, an indication that Turi’s recommendation reflects traditional knowledge shared with other populations to the east [36]. The ways in which Turi suggests to use frogs—i.e., either to directly rub a live frog on the affected area or to ingest a frog dried and cooked in milk—suggest the presence of bioactive compounds in frog skin. In the 1980’s it was discovered that frogs secrete antimicrobial peptides in their skin [37,38]. These peptides inhibit the growth of bacteria and fungi, and induce osmotic lysis in protozoa [39]. After the initial discovery of these peptides in the frog species *Xenopus laevis*, extensive research has been conducted to characterize the peptides found in other frog species, with the resulting finding that nearly all species secrete this class of peptides on their skin, but with clear variations in quantity and type according to species [40]. Turi insists that the frogs used should have white markings. Furthermore, if the frog used is “ugly” and bears black markings, sickness and even death can result. The official discovery of antimicrobial peptides in frogs occurred with a chance observation by a geneticist in the 1980’s, but perhaps a guided investigation of folk medicine efficacy could have yielded this result much sooner.

#### Ethnobotanical lore

Turi’s ethnobotanical lore is summarized in Table 2. The symptoms of earth-bostta, itchy scabs covering the body, coupled with Turi’s general suggestions to avoid contact with those suffering from the disease, suggest that earth-bostta is a type of infectious skin disease. Exact identification of the condition is difficult, however, since the term *bostta* in Sami could refer to a wide variety of ailments. Turi’s remedy calls for rubbing the affected areas of the body with a piece of **sod**, gathered from the bank next to a river or stream. It is probable that the sod mentioned could have contained lichens, some of which contain compounds useful as antibiotics. It may be important to note that usnic acid, a compound with established antiviral, antimicrobial, anti-proliferative, antiprotozoal, anti-inflammatory, and analgesic activity, is abundant in several widespread lichen genera [67,68]. Research activity related to usnic acid was especially abundant in the 1950’s, and then slowly decreased as some of the limitations of the acid’s isolation made it less cost-effective relative to other synthetic antibiotic drugs [82]. In the present day, the rise of multiple resistance microorganisms may lead to an increased interest in compounds like usnic acid. If appreciable amounts of usnic acid were available through the application of Turi’s treatment, then the potential action and efficacy can be readily perceived.

The plant ***Ferula assafoetida*** has a long and well-documented history as a source of culinary and medicinal compounds. Antifungal, antispasmodic, anti-diabetic, anti-inflammatory, and anthelminthic activities have been reported from an oleo-gum-resin obtained from the roots of this plant [62]. Turi recommends feeding sulfur powder and a component of *Ferula assafoetida* to dogs to cure “dog sickness.” The symptoms of this “dog sickness” that Turi reports strongly suggest that the underlying disease is a form of intestinal parasites. Pure sulfur powder has been used as a medical tonic and laxative [78]. Further support of the role of *Ferula assafoetida* as an anthelminthic can be seen in light of a recent patent application [64]. The application details the use of *Ferula assafoetida* resin to expel parasites in several animal species, including dogs. So by combining the laxative effect of pure sulfur powder with the anthelminthic properties of *Ferula assafoetida* Turi’s remedy may have indeed proven effective. It is important to note that both *Ferula assafoetida* and sulfur were available to Sami largely as trade goods rather than as substances readily gathered locally.

#### Mineral and chemical

Turi makes extensive recommendations concerning chemicals and minerals as healing agents, as shown in Table 3. Turi suggests ingesting powdered “fox gold” to treat joint pain. Available texts identify “fox gold” as muscovite, a mineral in the **mica** family [76]. Deposits of the mineral are common wherever igneous and metamorphic rock are found. It has been prized as a window-making material in Russia and as a mechanical lubricant [77]. Given that the mineral has a low solubility in acid and is relatively unreactive [77], it does not appear that muscovite would have any significant effect on the body when ingested in small quantities. A more interesting explanation may be rooted in mica’s use as a mechanical lubricant. It may be that Turi’s remedy for joint pain is an example of a sympathetic remedy. If mica was used in Fennoscandia as a mechanical lubricant for joints and junctions, folk healers may have wanted to apply the compound to improve the functioning of human joints.

Turi uses flecks of **copper** mixed in fat to ease swelling. Presumably the copper and fat mixture serves as an antimicrobial agent. Using copper in this capacity was widespread in the healing traditions of a variety of ancient cultures. The Egyptians, Aztecs, Persians, Greeks, and Romans used copper or copper derivatives (copper oxides, copper carbonate, and or copper acetate) to treat ear, eye, throat and wound infections in addition to a plethora of other ailments. From the Smith papyrus (ca. 2400 B.C.), Egyptian healers prescribed a remedy of (likely) copper carbonate mixed with grease to treat infected chest wounds [48].

Interest in the use of copper and related compounds for its healing properties has increased in more recent times as well. During nineteenth-century cholera epidemics in Paris, it was noticed that copper industry workers had a mortality rate ten to forty times lower than that of workers from other industries [48]. This may suggest that the copper in their work environment provided added immunity against the bacterium responsible for cholera, which was endemic at the time. Presently, clinical studies are being conducted to test the potential benefits of adding copper surfaces to hospitals and other settings where the risk of bacterial infection is high. Results from these studies show that copper is indeed effective in reducing the microbial load on commonly used hospital surfaces compared to aluminum or plastic control surfaces [49].

The exact mechanism though which copper exerts its antimicrobial actions has not fully been elucidated, but several research groups have proposed and demonstrated potential mechanisms. One of the most recent (2012), states that copper ions likely first cause bacterial cell membrane leakage and then protein oxidation and DNA degradation [50]. Turi’s remedy, therefore, could well have proven effective.

#### Conditions

The conditions and remedies provided by Turi are listed in Table 4. Turi’s texts indicate that he understood the basics of contagion. In explaining the remedy for certain illnesses, Turi will sometimes also mention what he believes to be the cause. He mentions that bodily excretions, odors, physical touch, and proximity contaminants can cause a variety of illnesses. For example, Turi states that one can develop old-maid bostta by experiencing a foul smell associated with old maids, or that pregnancy cravings can result from eating out of the same bowl as a pregnant woman.

Ideas of contagion were common among the Sami people, who believed that the maintaining the health of the body required the constant staving off of outside polluting forces like cold, heat, water, and human sweat [7]. Turi believed that wounds were serious, not only because of the associated tissue damage, but also because they could offer a path of entry for these polluting forces into the body. He offers several charms along with his physical remedies to help guard wounds against contamination. This view of contagion had direct consequences for how the Sami handled disease treatments. The Sami were reluctant to be in close contact with sick individuals, and healers took specific protective measures like wearing glasses to protect their eyes from contamination. Sköld has suggested that these practices limited the outbreaks of infectious diseases like smallpox among the Sami, helping explain differences in demographic data regarding the fatality of the disease [94].

#### Regimens

Turi’s healing regimens are summarized in Table 5. It is interesting to note that Turi provides accompanying charms for some of his treatments with the comment that they are not essential to the function of the treatment but can still improve the efficacy. While describing the treatment for skin eruptions (rubbing a frog on the affected area) Turi mentions that the Sami recite a charm while doing this, but that the remedy is still effective even without these words. Or in another case, after Turi says how to treat hemorrhage in childbirth (boiling *Delichon urbica* nest litter in milk and giving the mixture to the mother to drink), he states that this treatment helps the problem even if one does not know any accompanying incantation, but that it is more effective when an incantation is recited.

Yet in other cases words represent the major component of Turi’s remedies with a physical substance as auxiliary or not present at all. Turi states that in cases of difficult labor during childbirth it helps for the mother to say the name of the father, however, the efficacy can be improved if the mother also consumes some of the father’s urine [1].

In Turi’s healing repertoire words and physical substances have varying degrees of power depending on the ailment, as is typical of many north European folk healing traditions [29].

Turi makes frequent use of physical regimens for treating ailments. In the case of frost-bite, Turi recommends to rub the affected limbs with subsurface snow, translated as “corn-snow,” to restore circulation. The rigidity and pebble-like consistency of this type of snow may have rendered it particularly effective as a device for massage, in a manner different from that of drier, softer, or flakier snow. Turi’s specification of the type of snow to be used in such healing was probably a useful clarification for Sami.

Turi discusses several cures for toothache. One of these in particular involves rubbing the muscles of the jaw, neck, and shoulders. Massage with a similar technique as described by Turi has been shown to increase blood flow, provide temporary pain relief, and reduce muscle tension [80,81]. Additionally, localized muscle tension, especially in the masseter, is commonly misconstrued as tooth pain. The current recommendations for treating pain of this type include muscle stretching and massage, much as Turi recommends [86].

#### Veterinary medicine

Turi’s veterinary lore is summarized in Table 6. Turi’s detailed descriptions of reindeer ailments reveal the care with which he organized his heretofore oral knowledge and his ability to present this knowledge clearly to an outside audience. The fact that the researchers were able to identify plausible diseases on the basis of Turi’s descriptions demonstrates the accuracy of his observations. In general, however, Turi is less able to treat ailments than he is to identify them. Because Turi’s herding experience involved herds of hundreds or even thousands of animals, Sami could not generally provide individualized treatment to specific animals, although on occasion, as Turi notes, single animals could be tied up and subjected to particular treatments. Turi notes the effectiveness of some herders in acting as midwives for reindeer during parturition, but here again, the size of reindeer herds at the beginning of the twentieth century would have limited herders’ abilities to assist every animal experiencing distress.

It is difficult to comment on the potential efficacy of Turi’s remedies for reindeer ailments, but it is clear that Turi was able to recognize and diagnose specific reindeer ailments that have clear parallels in present-day veterinary medicine [88-90]. For example, the condition that Turi names as *“livzzavihki,”* characterized by emaciation and sagging of the rear end of the animal, finds a good match in parasitic infection by the nematode *Elaphostrongylus rangiferi*[91]. During the progression of this parasitic infection, this type of nematode reaches maturity in the shoulder and hind-limb muscles of the affected animal causing degradation of the muscle tissue and thus the sagging rear end. Somewhat surprisingly, despite living at such high latitudes, reindeer are exposed to a diverse collection of parasites and diseases. Turi’s knowledge of these illnesses with their corresponding remedies, though hard to decipher in terms of efficacy, do speak to his knowledge of reindeer physiology.

It should be noted that no appraisal of the overall clinical effectiveness of Turi’s knowledge is possible based on literature review alone. A complete assessment of Turi’s material in relation to this question would require a systematic testing of each of Turi’s methods in the laboratory.

### The degree to which magic is used in Turi’s remedies

**Figure 3:**
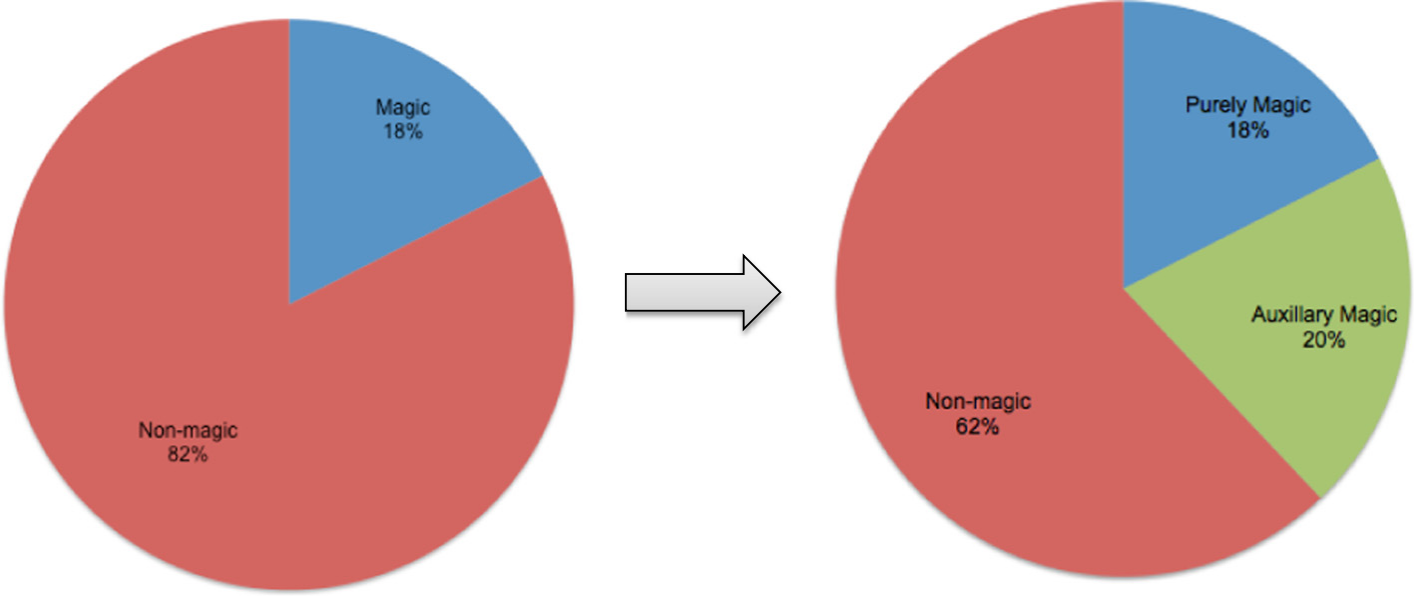
Magic in Turi’s remedies.

In Figure 3, “purely magical” treatments refer to acts that involve no other potential source of efficacy other than the proper performance of consciously articulated magical words or actions. For example, Turi describes how one can recite an incantation of sorts to accelerate the healing of wounds caused by iron weapons. For the purposes of this figure, this is considered a “purely magic” remedy. In many of Turi’s descriptions of healing methods, however, magic words or procedures are combined with the provision of particular plant or animal substances or in conjunction with specific physical acts, such as massage or application of heat. When all of these combined methods are summed, the percentage of healing acts linked in Turi’s view with magic rises to 38 percent.

### The degree to which Turi’s material reflects uniquely Sami knowledge or shows the influences of neighboring cultures and medical traditions at the outset of the twentieth century

In describing his healing knowledge as a whole, Turi states that Sami developed their medical traditions through a long process of trial and error. He writes:

"The Sami in past times had to figure out what to do when people got sick and there were no doctors living in the places where the Sami live, and some people didn’t even know that doctors exist. And so, they had to figure things out so much that they discovered what different ailments were like and what one needs to do to help them. And indeed they discovered so much that they could cure many diseases, even ones that many doctors cannot figure out how to cure. But this is not the case with every doctor. And here below one can find explanations for how to lessen each ailment and even cure some, and quickly too—not even a doctor could heal so quickly" [1] (T1: 119).

Turi’s statements show a clear awareness of official medicine, which had recently become available to Sami living in Turi’s home district through the establishment of medical services at Vittangi, a predominantly Finnish-speaking market town some sixty kilometers east of Turi’s home village of Jukkasjärvi. A midwife’s practice had been established at Vittangi in 1897, with a Swedish female practitioner. A male district physician had been added in 1901 [87]. In his descriptions of Sami healing, Turi mentions local people who have occasionally taken advantage of these new services, but only once other avenues of treatment had been exhausted [7]. His writings show an awareness of dominant medical discourse emanating from the Swedish government at the time castigating folk reliance on traditional healing and insisting on public embrace of official medicine. Turi seeks to justify his knowledge as having been developed in a period before official medicine had become available.

In ascertaining the typicality of Turi’s remedies in comparison with other accounts of Sami healing, the researchers made particular use of Just Qvigstad’s synthetic work *Lappische Heilkunde*[12], as well as a more recent study by Svanberg and Tunón [46]. Turi’s descriptions of moxibustion, for instance, are similar to those of other Sami healers of his era, and descriptions attest to Sami practice of moxibustion already in the eighteenth century. From these sources it was possible to determine that most of the remedies which Turi describes were known to some extent by other Sami healers of his era or later, although perhaps not with the same emphases or particular practices as Turi describes. In addition, the researchers examined Turi’s healing arsenal in relation to other North European and Russian folk healing, as presented in studies on medieval and later healing traditions [13,17,18,56,63,69,95,96], and in relation to data on traditional Sami diet [57-60,70]. Such comparison allowed the researchers to glimpse the possible vectors of healing knowledge into Sami culture at this time and to ascertain what aspects of Turi’s arsenal appeared unique to Turi or to Sami culture in particular. Turi’s recommendations for the practice of bleeding or moxibustion reflect the diffusion of European and Asian healing traditions into the Nordic region, most probably through a combination of official and folk healing.

In practice, however, the researchers came to realize that examination of Turi’s remedies with an eye to their native or imported nature imposes artificial boundaries on the data: for Turi, all the treatments described in his compendium were “Sami,” even though some of them show the influences of foreign healing traditions. The researchers found relatively few remedies that were not paralleled somewhere else in the world, although co-occurrence of a single treatment in two different geographic or cultural areas does not necessarily imply a process of cultural diffusion. Nonetheless, the researchers also noted some interesting instances of Turi’s awareness of medical borrowing, particularly in the area of magic formulas. Many of the formulas Turi supplied in fact, were borrowed from Finnish practitioners in his area, and Turi seems at least sometimes to have translated his magic words into Sami solely for the benefit of explaining them to Demant Hatt, who knew no Finnish [7]. The fact that magical knowledge apparently diffused here from Finnish culture into Sami is not a development that Turi’s editor Demant Hatt had expected to find, given that Sami were viewed by ethnographers of the time as more “primitive” than their cultural neighbors and therefore seemingly more prone to indulging in magic thought. The evidence, however, points in the opposite direction.

## Conclusions

Texts such as Johan Turi’s compendia of knowledge offer valuable glimpses into the healing traditions of an indigenous Sami man at a specific moment in time. Such texts demonstrate the importance of in-depth interviews with single informants as a balance and supplement to broader, potentially more superficial surveys. When in-depth data collection allows a knowledgeable healer to present materials in the way that the healer chooses, additional insights are gained: remedies may surface that the researcher had not expected to find, and the healer’s own categories of classification or interpretation become palpable in the presentation of the material. Affording informants the opportunity of producing a longer work can be time consuming and difficult, both in terms of editing and translation, but it can yield data of great value to researchers in the present and future. Johan Turi’s collaboration with Emilie Demant Hatt resulted in a wealth of recorded knowledge that continues to shed valuable light on the workings of Sami healing traditions even a century later.

## Competing interests

The authors declare that they have no competing interests.

## Authors’ contributions

TD provided source material and ethnographic expertise related to Sami language and culture. JL performed an extended literature review to evaluate the potential efficacy of Turi’s remedies. Both authors made substantial contributions to the project design and analysis. Both authors read and approved the final manuscript.
